# Mobility Network-based Measurement of Local Collective Efficacy and its Consequences for the Spatial Patterning of Violent Crime

**DOI:** 10.1007/s10940-025-09624-8

**Published:** 2025-08-12

**Authors:** Catherine A. Calder, Nicolo P. Pinchak, Christopher R. Browning, J. Brandon Carter, Emily Hsiao, Bethany Boettner, Jake Tarrence, Paul Bellair

**Affiliations:** 1 Department of Statistics and Data Sciences, University of Texas at Austin, Austin, TX 78705 USA; 2 Crown Family School of Social Work, Policy, and Practice, University of Chicago, Chicago, IL 60637 USA; 3 Department of Sociology, The Ohio State University, Columbus, OH 43210 USA; 4 Institute for Population Research, The Ohio State University, Columbus, OH 43210 USA; 5 Office of Forecasting Research and Analysis, Oregon Department of Human Services, Portland, OR USA

**Keywords:** Collective efficacy, Violent crime, Spatial models, Point processes, Neighborhoods

## Abstract

**Objectives:**

Estimate the extent to which local variation in collective efficacy, a measure of social cohesion and norms toward intervention among individuals, is associated with the sub-neighborhood spatial patterning of violent crime in Columbus, OH.

**Methods:**

Using estimates of local collective efficacy derived from survey data on individuals’ perceptions of collective efficacy in the neighborhoods and at their routine activity locations collected as part of the Adolescent Health and Development in Context Study and incident-level, point-referenced crime data from the Ohio Incident-Based Reporting System, we fit inhomogeneous Poisson process models.

**Results:**

We find that net of neighborhood-level collective efficacy, a one standard deviation increase in deviation in the local collective efficacy score from the neighborhood average local collective efficacy score is associated with a decline in the violent crime intensity by a factor of 0.858.

**Conclusions:**

Affirming Jane Jacobs’ arguments about informal social control dynamics at fine-grained levels, our study illustrates that collective efficacy operates and can be measured at sub-neighborhood levels across the entirety of a city. Our findings highlight the need to measure social processes at finer spatial scales in order to understand their effects on crime. Future research should prioritize collecting additional fine-grained data on social processes using individuals’ perceptions of the locations they frequent as part of their everyday mobility patterns.

## Introduction

Nearly a century of research has considered how individuals and cities can be organized to deter crime. Early work focused specifically on neighborhoods, finding that those with higher rates of racial heterogeneity, residential turnover, and poverty tend to experience higher rates of crime (Shaw et al. 1929; Shaw and McKay 1942; Park et al. 1984). Individuals within these neighborhoods, it was proposed, are hindered in their capacity to foster the norms and relationships necessary to regulate crime (Kornhauser 1978; Bursik and Grasmick 1993). Consistent with these expectations, contemporary studies find that crime tends to be lowest in neighborhoods characterized by high levels of collective efficacy, a construct capturing the extent of social cohesion and norms toward intervention among residents (Sampson et al. 1997; Teitler and Weiss 2000; Sampson 2012).

Due to the relevance of neighborhood factors in explaining within-city variability in crime, statistical analyses examining the association between social factors and crime are typically performed at an areal unit level. That is, crime counts are summed over geographic regions partitioning the study region (e.g., census tracts or block groups) and standard regression models for count data are used to quantify the association between crime counts and socio-economic characteristics of the areal unit derived from census data products or from surveys administered to a representative sample of residents of the areal unit. For example, in one of the most extensive data collection efforts in support of the study of neighborhoods and crime, Peterson and Krivo obtained counts of the FBI’s crime index offenses from 1999-2001 for 9,593 census tracts in 91 cities in 64 metropolitan areas across the US, as well as socio-economic, mortgage lending, and labor market structure data at the tract level (Peterson and Krivo 2000, 2010).

While the neighborhood, typically operationalized as a census tract or block group, has been the predominant geographic resolution of empirical studies of crime over the past several decades, recent work has noted there is substantial variability in crime within neighborhoods (Groff et al. 2010; Taylor 2015; Schnell et al. 2017). For instance, studies demonstrate that a very small number of locations (i.e., “hot spots”) can account for the majority of crimes in a city (Brantingham and Brantingham 1999; Weisburd et al. 2012; Schnell et al. 2017). Moreover, crime occurrences – when considered as points in a well-defined region (e.g., a city) occurring at precise moments in time – have been shown to resemble a contagion-like process (Johnson 2008; Bernasco and Nieuwbeerta 2005; Tita and Ridgeway 2007). In order to identify the location of crime hot spots and produce predictions/forecasts of future crime, researchers have developed statistical models based on a self-exciting spatio-temporal point process framework (Mohler et al. 2011).

Instead of using point process models for predicting the spread of crime, we return to the cross-sectional setting where the interest is in quantifying the association between crime occurrence and fine-scale, spatial-structured social processes. Specifically, we draw on routine activity theory and the work of Jane Jacobs to argue that collective efficacy is an inherently spatially continuous process, and one that implicates perceptions of all individuals carrying out routines in an area alongside those of residents—the sum of perceptions in the eyes of potential guardians, targets, and offenders. This argument suggests that collective efficacy varies spatially not just as a function of variability in reports and where people live, but also what both residents and nonresidents perceive as they move about an urban area.

We leverage point-level crime incident data for the city of Columbus, OH and point-level reports on levels of collective efficacy at routine activity locations (including home) provided by participants in the Adolescent Health and Development (AHDC) Study (Boettner et al. 2019). Instead of residential-based reporting on collective efficacy, we take advantage of the mobility network of AHDC participants to measure collective efficacy at a fine resolution. Since collective efficacy reports are not available at all locations in the study area, we generate local collective efficacy scores using the land-use filtering method introduced by Carter et al. (2024). We then use inhomogeneous spatial Poisson process models, with a parametric intensity function depending on local collective efficacy and other spatially-varying factors, to model the spatial patterning of violent crimes that occurred in the AHDC study area during the first wave of data collection. We find a statistically significant association between within neighborhood deviation in local collective efficacy and violent crime after accounting for variation in neighborhood average local collective efficacy and population size, as well as standard socio-demographic controls.

From a spatial methodology perspective, this paper illustrates how two novel sources of point-level spatial data, crime incidences and routine activity-based collective efficacy reports, can be used to study spatial variation in crime at a fine spatial scale. Unlike other studies of within-neighborhood crime, our analyses are based on data collected across a large city as opposed to small areas of cities. Our contribution also includes a discussion of the use of land use in generating accurate local collective efficacy scores and the consequences of doing so for confounding adjustment in downstream analyses of crime. While we do not focus on temporal forecasting of crime here, we note that the methodology could possibly be extended to this setting using, for example, tools developed by Reinhart and Greenhouse (2018).

## Background

We begin by reviewing the literature on neighborhoods and crime with a particular focus on the growing relevance of urban mobility in the crime and place literature. We then discuss the implications of this mobility for both the measurement of collective efficacy and its relation to crime at geographic scales below the neighborhood level. Finally, we discuss research on the interplay between collective efficacy and routine activities, which motivates the expectation that collective efficacy as perceived by resident and nonresident locals within an area contributes to crime control among residential and nonresidential locations.

### Urban Mobility and Crime

The mobility of residents and nonresidents within urban neighborhoods has long been suggested to be important for understanding spatial variation in crime. For example, McKay (1949, p. 32) conceived of the neighborhood as “the world of the child exclusive of his family,” including routine activity locations related to family, school, work, and leisure. Likewise, Jacobs (1961) observed that neighborhood spaces that routinely bring residents and nonresidents together facilitate “casual, public contact at the local level—most of it fortuitous, most of it associated with errands” which promotes norms about crime deterrence. True to this conception of neighborhood safety, Sampson et al.’s (1997) foregrounding study of neighborhood collective efficacy, the Project on Human Development in Chicago Neighborhoods (PHDCN), explicitly prompted respondents to factor routine activity locations into their collective efficacy reports, stating: “By neighborhood, we mean the area around where you live and around your house. It may include places you shop, religious or public institutions, or a local business district. It is the general area around your house where you might perform routine tasks, such as shopping, going to the park, or visiting with neighbors” (Earls et al. 2007, p. 3). Sampson and colleagues have also called attention to the need for data on experiences at specific locations to comprehensively measure urban social organization. For example, in explaining why, contrary to their hypothesis, a neighborhood-level measure of residents’ perceptions of local organizations was not associated with neighborhood crime, Morenoff et al. (2001, p. 553) suggested that residents may not reliably be aware of the full extent of local organizations, concluding that “independent data are needed on the number and type of organizations, along with their geographical distributions.”

Notwithstanding this long-held relevance of routine activities and their locations, only recently has high-resolution activity data become widely available (Browning et al. 2021). For example, drawing on geotagged data from nearly 400,000 Twitter users across 37 cities, Levy et al. (2020) found that neighborhood rates of poverty in the places residents go as well as where a neighborhood’s “outsiders” reside are associated with neighborhood homicide rates independent of neighborhood residential poverty rates. Twitter data have also been used to investigate how changes in the ambient population of neighborhoods are consequential for spatial variation in crime within neighborhoods over time (Malleson and Andresen 2015; Hipp et al. 2019). Considering evidence that Twitter data has limited reliability for capturing ambient populations especially in primarily residential areas (Tucker et al. 2021), researchers have used a variety of other mobility data sources such as the US Census Origin-Destination Employment Statistics (Graif et al. 2021; Kelling et al. 2021) and various cell phone-based data brokers (Massenkoff and Chalfin 2022; Sampson and Levy 2022) to investigate how mobility patterns relate to spatial variation in crime.

This growing literature underscores that the structure of everyday mobility flows between and within urban areas are essential for understanding spatial crime patterns. However, whether differential levels of social processes long thought to arise from these mobility flows across urban areas—such as the extent of cues of physical and social disorder, social network ties, and *collective efficacy* (Jacobs 1961; Kelling and Wilson 1982; Sampson 2012, p. 371)—are consequential for crime at micro scales has not been thoroughly investigated.

### Urban Mobility and the Scale of Collective Efficacy

Collective efficacy refers to the capacity of community members to work together to solve problems (Sampson et al. 1997). It is typically measured by combining responses to survey questions about the extent of social cohesion and willingness to intervene among locals, which are aggregated within census-based “neighborhood” units. In their influential test of collective efficacy theory from the PHDCN, Sampson et al. (1997) demonstrated that neighborhood-level collective efficacy partially mediated associations between neighborhood-level concentrated disadvantage and neighborhood violence in Chicago. In the quarter century since, replications in cities around the world have documented evidence that neighborhoods higher in collective efficacy experience lower crime rates (Wickes and Lanfear 2025), and some city governments even continuously track neighborhood levels of collective efficacy as part of their public safety monitoring efforts (e.g. in London; Sutherland et al. 2013).

Significant within-neighborhood variation in both crime and individuals’ everyday mobility highlights the question of whether social processes like collective efficacy operate at finer spatial scales. For example, Weisburd et al. (2012) suggested that collective efficacy could be stimulated at hotspots to deter crime, while others have contended that collective efficacy operates solely as a community-level process and that measurement of more crime-proximate processes at the micro-level—such as the presence of criminal opportunities or crime-prevention capacities of place managers—may be more pertinent for understanding the spatial distribution of crime (Braga and Clarke 2014; Eck and Madensen-Herold 2018). Despite such challenges, however, the potential for individuals routinely inhabiting an urban setting to be valid reporters of informal control norms at locations has long been acknowledged in urban research. In particular, Jacobs (1961) elaborated how trust can be fostered at the level of sidewalks to regulate crime. Commenting on its origin, Jacobs (1961, p. 56) stated: “The trust of a city street is formed over time from many, many little public sidewalk contacts,“ such as when shopkeepers give advice to patrons, gossip is exchanged while waiting in lines, or nighttime security is reinforced by networks of delivery workers, doormen, and dog walkers Jacobs (1961, p. 36-36, 40-41, 56-56). The summation of these interactions, she proposed, is an “almost unconscious assumption of general street support when the chips are down—when a citizen has to choose, for instance, whether he will take responsibility, or abdicate it, in combating barbarism or protecting strangers.” Hence, experiences of residents, patrons, workers, and other locals positioned to take on place management roles are key in efforts to comprehensively capture the social organization of urban spaces.

Aligning with Jacobs’ observations, research finds that neighborhoods characterized by more opportunities for “public contact” tend to have higher levels of collective efficacy (Jacobs 1961, p. 56; Browning et al. 2017b) and less crime (Browning et al. 2017a; Pinchak et al. 2023). Beyond the neighborhood level however, Jacobs’ observations suggest that collective efficacy can be fostered, expressed, and perceived by routine locals at the micro-level (e.g., “sidewalks”). Providing initial evidence for this proposition, Weisburd et al. (2020) found that residents near crime hot spots in Baltimore report lower levels of collective efficacy and higher levels of physical disorder than those of non-hot spots. Weisburd et al. (2021) furthermore found that collective efficacy at the street segment-level is associated with reduced street segment-level crime. These tests are restricted to perceptions among individuals living in small and largely residential areas, however, leaving open the question of whether collective efficacy reports by nonresidents at specific locations across the entirety of an urban area are also consequential for crime. This is undoubtedly due to the lack of available data on individuals’ experiences at their routine activity locations.

### Collective Efficacy, Urban Activity Locations, and Crime

Informal social control is a product of everyday interactions among locals, and these interactions are heavily shaped by features of the environments where they take place. This is a tenet of Jacobs’ *The Death and Life of Great American Cities*, which set in motion a diverse array of research programs examining how features of urban settings either reinforce or disrupt a host of urban social problems (Newman 1972; Jeffery 1971). Consistent with Jacobs’ expectations, studies find that many nonresidential spaces within neighborhoods can serve as “social conduits” by increasing interaction among local residents and routine nonresidents (Corcoran et al. 2018; Jacobs 1961). For instance, residents more commonly interact and encounter one another in neighborhoods with more shops, cafes, recreational facilities, and religious institutions (Lund 2003; Simões Aelbrecht 2016). Wickes et al. (2019) similarly find that social cohesion and trust among residents tend to be higher in neighborhoods with a greater prevalence of “anchoring conduits,” including schools and childcare centers, libraries, gyms and health clubs, community clubs, and religious institutions. Furthermore, Corcoran et al. (2018) finds that the density of social conduits in a neighborhood is associated with greater collective efficacy. These studies are largely focused on residents’ perceptions of collective efficacy across the entirety of their neighborhood, however, leaving open questions about whether nonresidents’ perceptions at such conduits contribute to collective efficacy and their consequences at within-neighborhood resolutions.

While acknowledging the potential crime-deterrent role of such nonresidential locations, there is also evidence that many of these increase crime. Particularly relevant in this respect is routine activity theory, which highlights how various features of the built environment facilitate crime through the differential convergence of motivated offenders, potential victims, and capable guardians (Cohen and Felson 1979; Felson and Boba 2010). Research in this tradition finds that many different types of urban locations provide opportunities for crime (Wilcox and Cullen 2018), such as when delinquent groups mobilize around schools (Steinberg et al. 2019), parks are used to deal drugs (Groff and McCord 2012), alcohol outlets stir fighting (Roncek and Maier 1991; Wheeler 2019), or vacant properties signal disorder (Taylor 1988) and are used to hide crimes (MacDonald et al. 2019). Similarly, social holes and wedges, such as industrial areas and parks, tend to reduce neighborhood cohesion and the likelihood that locals interact with one another (Hipp et al. 2014) and can sometimes increase crime in nearby areas (Groff and McCord 2012; Boessen and Hipp 2018).

The potential for social control capabilities to develop at purely nonresidential locations is well-acknowledged in the literature. In addition to Jacobs (1961)’s observations, Cook and MacDonald (2011) found that business improvement districts – areas where businesses pay additional tax to, among other things, increase security and reduce disorder – reduce crime in Los Angeles. Cui et al. (2022) additionally found that a vacant lot greening initiative reduced crime in Philadelphia, particularly when implemented near civic institutions, convenience stores, or pharmacies as well as residential areas. Consistent with these results, studies find that a primary way neighborhood-level collective efficacy reduces crime is by facilitating removal of land use and built environment-related criminal opportunities, such as vacant properties and industrial sites (Zahnow et al. 2021; Lanfear 2022). Nevertheless, whether collective efficacy is related to reduced crime at small scales beyond what is perceived by individuals in their immediate residential environment has yet to be investigated.

In sum, individuals’ perceptions of their routine activity settings both residential and nonresidential have long been thought to be essential for measuring collective efficacy and its relation to crime at fine-grained levels. Innovative sampling strategies offer evidence that residents’ perceptions of collective efficacy are associated with reduced crime at the level of street segments (Weisburd et al. 2020), but the extent and consequences of collective efficacy across the entirety of an urban area has yet to be captured. Here we do so with perceptions of collective efficacy reported at routine activity locations by a representative sample of participants in the AHDC study. Specifically, these point-level data are used to measure collective efficacy as a spatially continuous process across all of Columbus, OH and consider whether within-neighborhood, point-level variation in collective efficacy is associated with point-level variation in violent crime. In an exploratory analysis, we also consider whether this within-neighborhood association of collective efficacy varies by areas designated as residential, commercial, and “other”.

## Data

### Adolescent Health and Development in Context Study

The AHDC Study is a longitudinal study of a representative sample of households designed to better understand the multiple contexts to which youth are exposed as they go about their daily routines and the consequences of these exposures for health and wellbeing. The study was conducted in the Columbus, OH metropolitan area.^1^

Our measure of local collective efficacy is derived from data collected as part of the AHDC Study. Participants in this longitudinal study are a representative sample of youth aged 11-17 and their primary caregivers who resided within the I-270 belt loop surrounding the city of Columbus, OH. We use reports caregivers provided about their routine activity locations during Wave 1 of the study (2014-2016). For each activity location, caregivers reported the location type (e.g. home, kid’s school, friends’ and relatives’ houses, work, grocery stores, etc.) and when they typically spend time at the location (e.g. daytime, nighttime, weekdays, weekends). They also answered several questions about their impressions of the social climate at these locations.

### Collective Efficacy Location Reports

Our measure of local collective efficacy was derived from 9,526 responses provided by 1,369 AHDC Wave 1 caregivers to at least one of three social climate questions (with 5,436 of the 9,526 including answers to all three questions) – corresponding to the informal social control (“defense"), social cohesion (“trust"), and monitoring (“observation") components of collective efficacy – at their routine activity locations^2^, of which 4,526 were unique. These questions were posed as statements to which the caregiver rated agreement on a five-point Likert scale: *If someone was being threatened near [location], other people around would come to their defense. You can trust people on the streets in the area near [location]. There are usually people watching what’s happening in the area near [location].* The response options to the first two statements were “strongly agree," “agree," “neither agree nor disagree," “disagree," and “strongly disagree," while the response options to the last statement were “never," “almost never," “sometimes," “fairly often" and "very often." These response categories were coded such that 1 corresponded to the least affirmative value (never or strongly disagree) and 5 the most affirmative value (very often and strongly agree) for all three components. We refer to the responses to these questions and their corresponding latent spatial processes, respectively, as “defense," “trust," and “observation." While location ratings outside of the study area were provided, we restrict our analysis to the responses from 4,526 unique locations within the I-270 belt loop. Of the 1,369 caregivers in the data set, the number of reports by a single caregiver ranged from 1 to 27, with an average of 6.8 reports. For more details on the AHDC collective efficacy reports, see Carter et al. (2024).

**Fig. 1:**
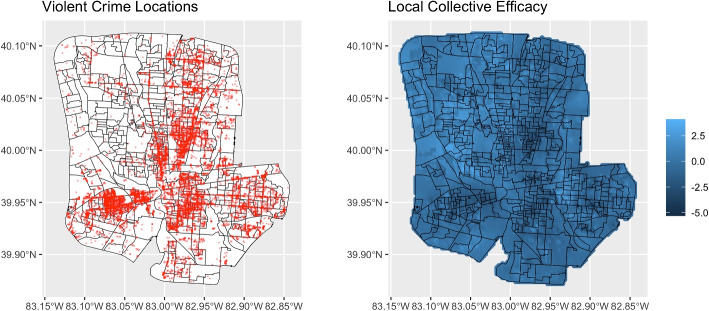
Left: 2014-2016 violent crimes occurring within the AHDC study area. Right: Derived local collective efficacy. Black lines denote block group boundaries

### Neighborhood Controls

In our statistical analyses, we control for several neighborhood-level variables known to be associated with both collective efficacy and crime. These controls are based on the U.S. Census Bureau’s American Community Survey (ACS) estimates encompassing the five year period preceding the start of AHDC Wave 1 data collection and are at the census block group level following Pinchak et al. (2023). The neighborhood control variables are: *percentage young males*, the number of resident males between the ages of 15–24 divided by the total population in the block group; *percentage of the population that identifies as Black*, *residential instability*, a standardized scale of the proportion of residents ages 5 and older who moved in the past 5 years and the proportion of occupied housing units that are renting; and *socio-economic status* (SES), the average of six block-group standardized variables including the proportion of residents with a college degree, the proportion of residents in a professional occupation, the proportion of households with at least $50,000 in annual income, the reverse coded proportion of residents in poverty, the proportion of households that are female headed, and the proportion of residents ages 16–64 who are unemployed.

### Crime Data

Incident-level, point-referenced crime data was obtained from the Ohio Incident-Based Reporting System (OIBRS) for Franklin County, OH for the period 2014-2016. For the analyses presented in this paper, we focus on violent crimes (robbery, homicide, aggravated assault, and rape) occurring in the AHDC study area. Our analyses are based on the locations of 11,778 violent crimes, including 4,117 assaults, 241 homicides, 1,758 rapes, and 5,662 robberies. The latitude/longitude coordinates (in the EPSG:3857 projection coordinate system) of the crime locations are shown in the left panel of Fig. 1.

## Methods

### Local Collective Efficacy Scores

For each component of collective efficacy (“defense," “trust," and “observation"), indexed respectively by $\ell = 1, 2, 3$, each AHDC caregiver provided a rating on a five-point ordinal scale, where a rating of 1 is the “worst" and a rating of $K\equiv 5$ is “best," for each of their routine locations. Following Carter et al. (2024), we define notation that indexes the local collective efficacy reports by location, not by the caregiver providing them. We let $\textbf{s}_i\in \mathcal {A}$ denote the *i*th location in the AHDC study area, where $i=1,\dots ,m$, and $Y_{ij\ell }$ denote the *j*, $j = 1,\dots ,n_i,$ rating of the *i*th location on the $\ell$ collective efficacy component. Thus, the complete set of collective efficacy component ratings are denoted by $\mathcal {Y} = \{Y_{ij\ell }: i=1,\dots ,m; j=1,\dots n_i; \ell =1, \dots , 3\}$.

As in Carter et al. (2024), we specify a spatial statistical model for $\mathcal {Y}$ using a well-known Bayesian specification of a probit model for ordinal data. Specifically, we introduce continuous latent variables $\textrm{Z}^*_{ij\ell }$ and assume that

$$ Y_{ij\ell } = {\left\{ \begin{array}{ll} K & \text {if } \gamma _{K-1,\ell }< \textrm{Z}^*_{ij\ell }\\ k & \text {if } \gamma _{k-1, \ell }< \textrm{Z}^*_{ij\ell }< \gamma _{k\ell }, \text { for } k = 2, \dots , K-1\\ 1 & \text {if } \textrm{Z}^*_{ij\ell } < \gamma _{1\ell } \end{array}\right. }. $$

We then assume that the $\textrm{Z}^*_{ij\ell }$s are conditionally independent given the value of another latent spatial process at location $\textbf{s}_{i\ell }$, denoted $\tilde{Z}_{\ell }(\textbf{s}_i))$. Importantly, the process $\tilde{Z}_{\ell }(\textbf{s})$ is well defined for all $\textbf{s} \in \mathcal {A}$, so we can generate predictions across the entire study area.

Our specification of a model for $\tilde{Z}_{\ell }(\textbf{s})$ is motivated and detailed in Carter et al. (2024). Briefly, the model allows for nonstationarity in the latent process using a technique that we refer to as “land-use filtering." This technique allows the mean, variance, and strength of spatial dependence of the spatial process to depend on the land-use categorization of parcels – “residential, “nonresidential," and “other" – as assigned in taxation records obtained from the Franklin County Auditor’s website. Carter et al. (2024) provides empirical support for leveraging spatial variation in land use to spatially smooth the collective efficacy reports across the study region in the form of exploratory analyses and model comparison, but does not argue about the direction of a causal relationship between the two.

Conditioning on the maximum *a posteriori* (aMAP) estimates of the corresponding parameters in an approximation to the land-use filtering model (see Section 3.5.2 of Carter et al. (2024)), we generate predictions of each local collective efficacy component $\tilde{Z}_{\ell }(\textbf{s})$, denoted $\hat{\tilde{Z}}_{\ell }(\textbf{s})$, on a fine, regular grid of points covering the study region, with points indexed by $\mathcal {G} = \{\textbf{u}_1, \dots , \textbf{u}_G\}$, where all $\textbf{u}_g \in \mathcal {A}$ and $G = 11,387$. Points on the prediction grid are approximately 5 meters apart, which was chosen to ensure predictions were available at a finer resolution than one might expect any meaningful variation in collective efficacy. For each grid point, *g*, we calculate

$$ \hat{Z}(\textbf{u}_g) = \frac{1}{3} \sum _{\ell = 1}^{3} \hat{\tilde{Z}}_{\ell }(\textbf{u}_g), $$

averaging over the three components of collective efficacy. We then compute standardized predictions, $\hat{Z}^{std}(\textbf{u}_g)$, of collective efficacy by subtracting the mean and then dividing by the standard deviation of the collective efficacy predictions over all $g \in \mathcal {G}$. The values of $\hat{Z}^{std}(\textbf{u}_g)$ are shown in right panel of Fig. 1, and we refer to them as the *local collective efficacy (LCE) scores*.

### Neighborhood and Deviation Local Collective Efficacy Scores

Unlike our LCE scores, collective efficacy has been traditionally operationalized at an aerial resolution coinciding with a particular census geography. For example, PHDCN participants’ neighborhood collective efficacy reports were aggregated within “neighborhood clusters” (groups of contiguous, homogeneous census tracts).

For our crime analyses in Section “Spatial Poisson Regression Models”, we define two additional quantities using the local collective efficacy scores. First, we define the *block-group average local collective efficacy score* for block group *m* as

$$ X^{\overline{lce}}_m = \frac{1}{\vert \{\textbf{u}_g: \textbf{u}_g \in \mathcal {A}_m\}\vert } \sum _{\textbf{u}_g \in \mathcal {A}_m} \hat{Z}^{std}(\textbf{u}_g), $$

where $\mathcal {A}_m \subset \mathcal {A}$ denotes the *m*th block group contained in the study area $\mathcal {A}$ and $\vert \{\textbf{u}_g: \textbf{u}_g \in \mathcal {A}_m\}\vert$ denotes the number of points in $\mathcal {G}$ contained in $\mathcal {A}_m$. Unlike our block-group average LCE scores, traditional areal-level measures of collective efficacy are obtained using multi-level models. In this setting, individual-level reports on neighborhood collective efficacy are nested within areal units (e.g., census geographies) treated as surrogates for neighborhoods. On the other hand, our land-use filtering approach has already accounted for the structure of the location reports, so we simply average LCE scores to create a block-group average.

Finally, we define the *deviation local collective efficacy (*$\Delta$*LCE) score* as the difference between the LCE score at grid point *g* and the $\Delta$LCE for the block group containing $\textbf{u}_g$. That is, the deviation local collective efficacy score is

$$ X^{\Delta lce}(\textbf{u}_g) = \hat{Z}^{std}(\textbf{u}_g) - X^{\overline{lce}}_{b(\textbf{u}_g)}, $$

where the function $b(\textbf{s})$ returns the index of the block group containing a point $\textbf{s} \in \mathcal {A}$.

### Spatial Poisson Regression Models

To model the relationship between the locations of crime and our derived local collective efficacy measure, we use a statistical model based on an inhomogeneous Poisson point process (PPP; Diggle 2013), which is sometimes referred to as a nonhomogeneous PPP. The inhomogeneous PPP on a spatial domain $\mathcal {A} \subset \mathbb {R}^2$ is characterized by an intensity function $\lambda : \mathbb {R}^2 \rightarrow [0, \infty )$, such that the integral of $\lambda$ over all subsets of $\mathcal {A}$, $B \subseteq \mathcal {A}$, is finite,

$$ \Lambda (B) = \int _B \lambda (\textbf{s}) d(\textbf{s}) < \infty , $$

and

$$ \text {Pr}(N(\mathcal {B}) = n_i; i=1, \dots , k) = \prod _{i=1}^k \frac{\left( \Lambda (B)\right) ^{n_i}}{n_i !} \text {exp}\left( \Lambda (B)\right) , $$

where $N(\mathcal {B})$ is the number of points contained in *B*. It follows that $\Lambda (B) = \text {E}\left[ N(B)\right]$.

Letting $\mathcal {A}$ denote the AHDC study area, we specify the log intensity function as:

**Model 1:** $$\text {log}\left( \lambda (\textbf{s})\right) = \beta _0 + \beta ^{pop} X^{pop} _{b(\textbf{s})} + \boldsymbol{\beta }^{c} \textbf{X}^{c} _{b(\textbf{s})}$$
**Model 2:** $$\text {log}\left( \lambda (\textbf{s})\right) = \beta _0 + \beta ^{pop} X^{pop} _{b(\textbf{s})} + \boldsymbol{\beta }^{c} \textbf{X}^{c} _{b(\textbf{s})} + \beta ^{lce} X^{lce}(f(\textbf{s}))$$
**Model 3:** $$\text {log}\left( \lambda (\textbf{s})\right) = \beta _0 + \beta ^{pop} X^{pop} _{b(\textbf{s})} + \boldsymbol{\beta }^{c} \textbf{X}^{c} _{b(\textbf{s})} + \beta ^{\overline{lce}} X^{\overline{lce}}_{b(\textbf{s})}$$
**Model 4:** $$\text {log}\left( \lambda (\textbf{s})\right) = \beta _0 + \beta ^{pop} \textbf{X}^{pop} _{b(\textbf{s})} + \beta ^{c} X^{c} _{b(\textbf{s})} + \beta ^{\overline{lce}} X^{\overline{lce}} _{b(\textbf{s})} + \beta ^{\Delta lce} X^{\Delta lce} (f(\textbf{s}))$$,

where $X^{pop}_{b(\textbf{s})}$ is the total population size of the block group containing location $\textbf{s}$, $\textbf{X}^{c} _{b(\textbf{s})}$ is a vector of values of the standardized control variables (see Section “Neighborhood Controls”) for the block group containing location $\textbf{s}$, $X^{lce}(f(\textbf{s}))$ is the standardized predictive collective efficacy score for the grid cell closest to the location $\textbf{s}$, $X^{\overline{lce}} _{b(\textbf{s})}$ is the average local collective efficacy score for the block group containing location $\textbf{s}$, and $X^{lce} (f(\textbf{s}))$ is the deviation local collective efficacy score at the grid cell closest to location $\textbf{s}$. To facilitate interpretation of the $\beta$ parameters, total population, the block-group controls, and local collective efficacy have been standardize to have mean zero and variance one prior to analysis.

Our inferences provided in the next section focus on the $\beta$s. To find the maximum likelihood estimates (MLEs) of model parameters we use the ppm function contained in the spatstat package for R (Baddeley et al. 2015). This function returns maximum likelihood inferences on model parameters using the Berman-Turner algorithm (Berman and Turner 1992). Holding parameters fixed at their MLEs, we can also estimate the log intensity surface of crime over the study area.

## Results

Table 1 provides a summary of the estimated regression coefficients in the four trend models listed in Section “Spatial Poisson Regression Models”, along with corresponding standard errors. P-values corresponding to one-at-a-time Wald-type hypothesis tests of the true coefficients being equal to zero are also given.

**Table 1:** Estimated coefficients from the fitted inhomogeneous Poisson process models with different trends specifications listed in Section “Spatial Poisson Regression Models”

	Model 1	Model 2	Model 3	Model 4
Intercept	2.952***	2.652***	2.424***	2.428***
	(0.029)	(0.030)	(0.031)	(0.031)
Total Population	${-0.570}$***	${-0.403}$***	${-0.269}$***	${-0.272}$***
	(0.018)	(0.018)	(0.018)	(0.018)
% Young Male	1.335***	1.605***	1.737***	1.745***
	(0.140)	(0.140)	(0.140)	(0.140)
% Black	0.235***	0.037	${-0.042}$	${-0.049}$
	(0.038)	(0.038)	(0.038)	(0.038)
Residential Instability	0.085***	0.112***	0.136***	0.135***
	(0.010)	(0.010)	(0.010)	(0.010)
SES	${-0.652}$***	${-0.399}$***	${-0.205}$***	${-0.209}$***
	(0.013)	(0.014)	(0.016)	(0.016)
Local Collective Efficacy		-0.468***		
		(0.009)		
BG Average Collective Efficacy			${-0.810}$***	${-0.800}$***
			(0.014)	(0.014)
$\Delta$ Local Collective Efficacy				${-0.153}$***
				(0.014)
AIC	$-47,295.0$	$-49,457.7$	$-50,282.4$	$-50,390.0$

+ $$p<$$ 0.1, * $$p<$$ 0.05, ** $$p<$$ 0.01, *** $$p<$$ 0.001

Focusing on Model 4 for illustration, $\hat{\beta }_0 = 2.428$ implies we expect $\text {exp}(2.428) = 11.336$ violent crimes per square kilometer in a three-year period when all covariates are set equal to zero (which is their mean since the variables are standardized). Similarly, for a one standard deviation increase in SES, we expect violent crime to decline by a factor of $\text {exp}(-0.209) = 0.811$.

Moving from Model 1 to Model 2, we see that LCE is negatively associated with crime intensity after adjusting for the controls. Aggregating the LCE predictor to the block-group, Model 3 confirms our expectation that the negative association between collective efficacy and log crime intensity holds at this resolution, consistent with expectations in the literature. Comparing Models 2 and 3 to Model 1, we see that including the point level or block-group level collective efficacy in the model affects the relationship between the controls and log crime intensity. For example, the magnitude of the SES coefficient declines by about one-third/two-thirds for Models 2 and 3, respectively, and the percent Black coefficient is no longer statistically significant. The block-group average local collective efficacy coefficient, however, is statistically significant which implies that for a one-unit increase in block-group average local collective efficacy, the violent crime rate is expected to decrease by a factor of $\text {exp}(-0.810) = 0.445$, holding all other covariates at their mean. From the Model 4 results, we see that a one standard deviation increase in the deviation local collective efficacy score is associated with a decline in the violent crime intensity by a factor of $\text {exp}(-0.153) = 0.858$.

**Fig. 2:**
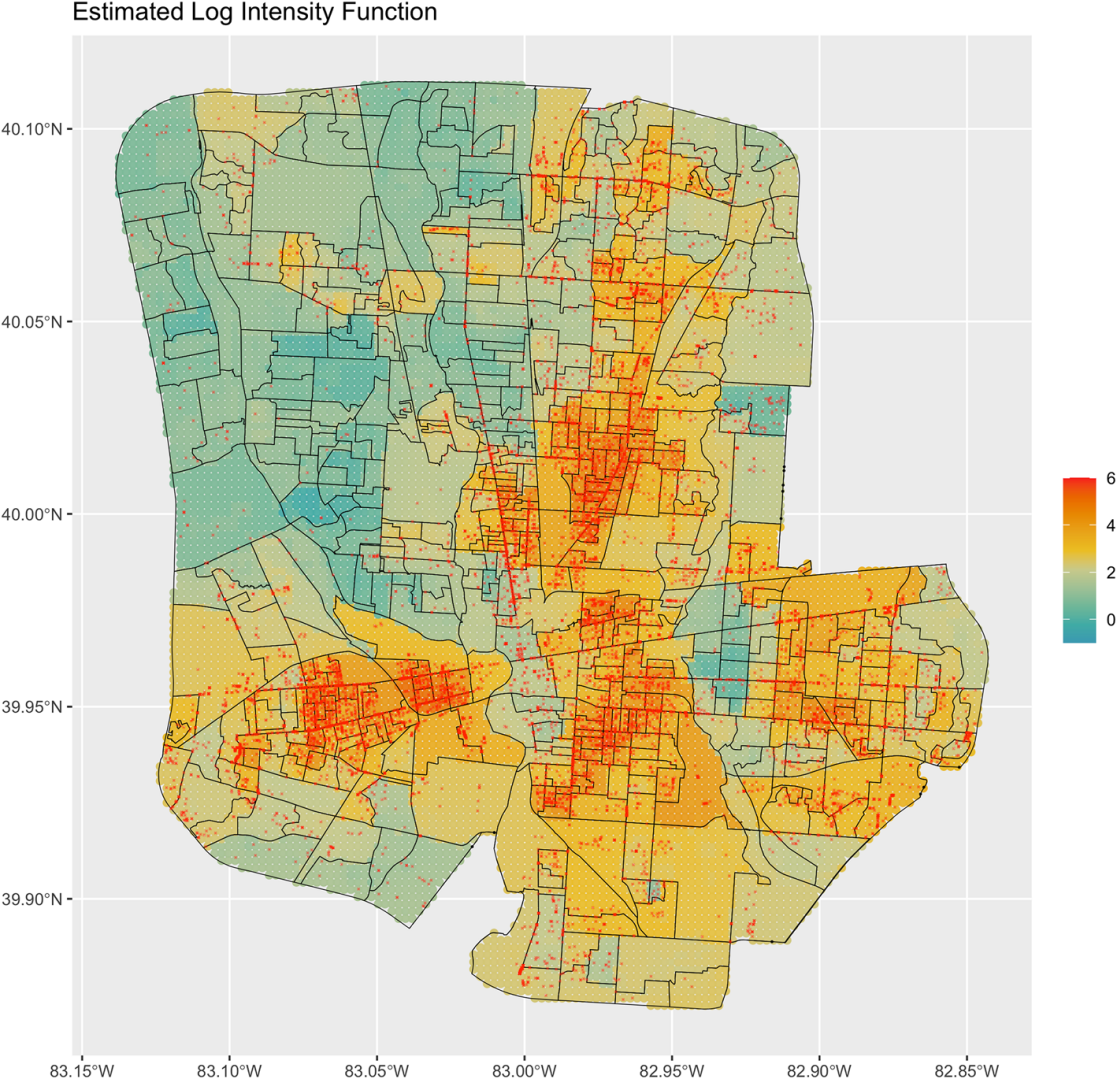
Model 4 estimated log intensity of crime over the AHDC study area. Black lines denote block group boundaries. Red dots are locations of violent crimes

Based on the AIC criteria, we find that Model 4 best fits the data. As a check of model fit, we plot the fitted log intensity function of crime over the AHDC study area in Fig. 2, with the violent crime counts shown in red. From this figure, we can see that variation in the estimated log intensity function shows considerable correspondence to the spatial patterning of crime location.

Finally, we note three limitations of our analysis. First, we do not account for the uncertainty in the LCE scores in our crime analysis. To the best of our knowledge, analytic methods for accounting for covariate measurement error in spatial point process models do not yet exist. While a multiple imputation (Monte Carlo) approach could be explored if uncertainty estimates associated with the scores were available, the approximate inference approach employed only returns point estimates. A second limitation is that our model does not account for spatial clustering (inhomogeneity) in crime not explained by covariates. While in theory it is possible to allow for such clustering via specification of a log-Gaussian point process within the spatstat package, the size of our data set made fitting such models infeasible. Lastly, we do not adjust for land use in our model for the log intensity function, $\log (\lambda (\textbf{s}))$. If land use in fact confounds the relationship between LCE and crime – that is, it has an effect on the log intensity independent of LCE – then failing to adjust for it will lead to biased estimates of the effect of LCE. On the other hand, statistical adjustment for confounding undoubtedly introduces a new source of bias, one that we refer to as *artificial collinearity*. In the following section, we explore this issue in more detail.

## Supplementary Exploratory Analyses of LCE, Land Use, and Crime

With nearly 10,000 collective efficacy reports, the AHDC Study provides unprecedented measurement of collective efficacy across an urban area. However, the spatially-referenced collective efficacy reports are not uniformly distributed across the study area. Instead, they reflect the day-to-day mobility of a representative sample of individuals. Naturally, the number of collective efficacy reports is higher in areas with popular destinations and where residential density is greater. The land-use filtering methodology used to obtain the local collective efficacy scores is well suited to accommodate the non-uniformity of the reports. In regions of the city with dense, consistent reports, the LCE predictions from the model are driven by nearby reports, a feature of the Carter et al. (2024) model. In other areas with sparse reports, land use, as a spatially-referenced covariate, more heavily drives the LCE predictions. An assessment of the “value” of the land-use filtering methodology in terms of predictive accuracy is explored in Section 4.2 of Carter et al. (2024). Comparisons to a simpler model (i.e., one with a stationary covariance structure) indicate that the land-use filtering methodology has superior predictive accuracy.^3^

We can view the LCE scores as noisy estimates of an unobservable “true” social construct that varies over space. Importantly, the scores are likely to be more correlated with land use than the “true” LCE process, a consequence of the land-use-driven spatial smoothing used to obtain them. Thus, a purely statistical limitation of our methodology for obtaining LCE scores is that we may introduce *artificial collinearity* in any downstream analyses where collective efficacy and land use are considered fixed and known (i.e., as independent variables). By “artificial," we emphasize that this is a source of additional collinearity that would not exist if we had perfect measurement of LCE. This artificial collinearity complicates downstream empirical analyses of crime involving both LCE (or $\Delta$LCE) and land use as covariates (e.g., to adjust for confounding). For example, consider an extension of Model 4 (say, Model 5) in which land use, as a categorical variable, is also included in trend. Standard interpretations of the of results may not be valid due to bias in the estimate of the effect of $\Delta$LCE scores resulting from artificial collinearity induced by using land use to improve predictions of $\Delta$LCE. Beyond collecting more, and spatially-denser, collective efficacy ratings across the study region, a costly and time-intensive endeavor, there is not a statistical solution to address this trade-off between the accuracy of the $\Delta$LCE scores and the potential bias in the crime analysis. That is, using land-use to predict collective efficacy appears to be beneficial in terms of improving predictive accuracy of the LCE scores, but comes at the potential cost of increased bias in the $\Delta$LCE score coefficient in the crime model.

With this disclaimer in mind and given the strong substantive interest in criminology about the role of land use in explaining the spatial patterning of crime, we present additional, exploratory analyses in Table 2. Model 5 extends Model 4 in Table 1 to include land-use as a categorical/factor variable in the log intensity function model, coded with “residential" as the baseline category. In addition, Model 6 includes the interaction between $\Delta$LCE scores and land-use category. From Table 2, we see that the effect of $\Delta$LCE remains statistically significant (Model 5) and is stronger (more negative) in nonresidential areas (Model 6).

**Table 2:** Estimated coefficients from the exploratory models described in Section “Supplementary Exploratory Analyses of LCE, Land Use, and Crime”

	Model 5	Model 6
Intercept	2.627***	2.627***
	(0.031)	(0.031)
Total Population	${-0.251}$***	${-0.250}$***
	(0.018)	(0.018)
% Young Male	1.268***	1.253***
	(0.140)	(0.140)
% Black	${-0.064}$+	${-0.060}$
	(0.039)	(0.039)
Residential Instability	0.168***	0.172***
	(0.011)	(0.011)
SES	${-0.293}$***	${-0.290}$***
	(0.016)	(0.016)
BG Average Collective Efficacy	${-0.704}$***	${-0.705}$***
	(0.015)	(0.015)
$\Delta$ Local Collective Efficacy	${-0.144}$***	${-0.084}$***
	(0.014)	(0.016)
Land Use (Nonresidential)	${-0.198}$***	${-0.215}$***
	(0.021)	(0.021)
Land Use (Other)	${-1.214}$***	${-1.219}$***
	(0.041)	(0.041)
$\Delta$LCE * LU (Nonresidential)		${-0.246}$***
		(0.032)
$\Delta$LCE * LU (Other)		${-0.012}$
		(0.092)
AIC	$-51,588.3$	$-51,641.5$

+ $$p<$$ 0.1, * $$p<$$ 0.05, ** $$p<$$ 0.01, *** $$p<$$ 0.001

To lend credibility to our exploratory analyses, we performed the following supplementary analysis. First, we identified block groups in the AHDC Study area with few collective efficacy reports, where somewhat arbitrarily “few" is defined to be less than two. The 91 census block groups (out of 615) that meet this criteria cover approximately 11 percent of the full study area. The LCE scores from the land-use filtering model over these block groups will be driven primarily by the spatial variation in land use due to the limited number of collective efficacy reports. Intuitively, there is no way to empirically assess whether the LCE scores, or the $\Delta$LCE scores, in these areas are artificially collinear with land use in a downstream analysis of crime.

On the other hand, for the subregion of the AHDC study area consisting of block groups with two or more collective efficacy reports, we have an opportunity to explore the extent to which artificial collinearity may be in fact a concern in a downstream crime data analysis with land use in the trend. Using only the collective efficacy reports from this “densely observed” subregion, we refitted the land-use filtering model (“original model”) and an alternative model (“global-intercept model”) in which land use is not included in the mean trend, but remains in the second-order components of the model to capture nonstationarity in the strength of spatial dependence and variance. Due to the complex, non-linear ways in which land-use drives the LCE predictions in the global intercept model, we expect artificial collinearity to be less of an issue if scores from this model were used in a downstream crime analyses with land use as a control. (As an aside, note that we actually would not want to use these global-intercept-model scores because the original model has better predictive accuracy: the root mean squared error (RMSE) for the global intercept model is 0.745 compared to 0.640 for the original model.) Instead, we compare the original model $\Delta$LCE scores to the global intercept model scores as an artificial-collinearity diagnostic. A higher correlation indicates that artificial collinearity is less of a concern in downstream crime analyses with land use as a control. On the other hand, a low correlation would not allow us to rule out the possibility that artifical collinearity is biasing downstream inferences. Lastly, we note that there is an inherent trade-off to consider in our diagnostic approach. It is unlikely that we would observe both a high correlation between the two sets of scores and a substantial improvement in predictive accuracy over the global-intercept model. That is, ideally there is a high, but not too high (i.e., “perfect"), correlation between between the two sets of scores.

**Fig. 3:**
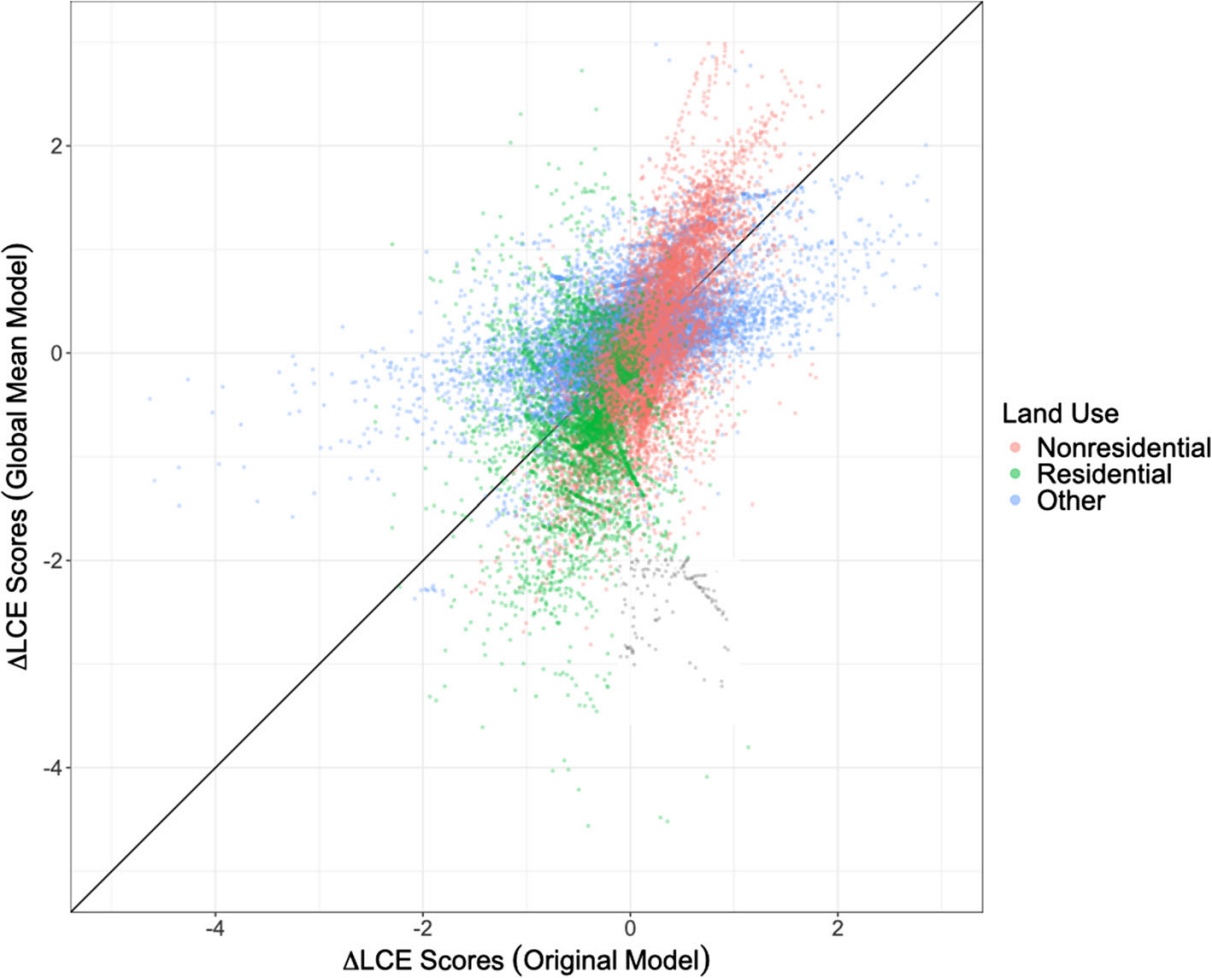
Deviation local collective efficacy scores from the original model and the global-intercept model by land-use category

Figure 3 compares the $\Delta$LCE scores from the global intercept model to those from the original model on fine grid over the subregion consisting of two or more collective efficacy reports (i.e., the “densely observed" area). The overall (across all three land use categories) correlation between the global-intercept model and original model scores is 0.50. The correlation for scores in residential areas is .54, similar to the overall correlation. The correlation is higher for the scores in nonresidential areas, 0.71, but only -0.07 in ‘other’ areas.

Unfortunately, we do not observe a particularly high overall correlation meaning that we cannot rule out artificial collinearity biasing our estimate of $\Delta$LCE coefficients. However, within nonresidential areas, the high correlation between the deviation LCE scores suggest that perhaps the $\Delta$LCE/land-use interactions involving nonresidential land use in Model 5 might be cautiously interpretable.

## Discussion

The concept of collective efficacy has proven to be an important contribution to understanding the community-level processes explaining spatial inequality in urban crime rates. The initial and highly significant findings stemming from the Project on Human Development in Chicago Neighborhoods (PHDCN) arguably constitute an apex of research and data collection quality and innovation in this area. Despite the compelling evidence of beneficial collective efficacy influence on crime emerging from the PHDCN project, few subsequent studies have extended measurement efforts in the nearly three decades since the publication of Sampson and colleagues critical findings (1997); although see O’Brien (2018) for a novel measurement strategy employing 311 call data from Boston.

At least three limitations characterize the now conventional approach to measuring collective efficacy as developed by Sampson and colleagues. First, the well-known “neighborhood cluster” approach to operationalizing residential neighborhoods employed in the PHDCN combines 2-3 census tracts maximizing internal demographic homogeneity and following significant ecological boundaries where possible. Questions about collective efficacy directed to PHDCN respondents, however, used the neighborhood definition previously cited in Section “Urban Mobility and Crime” which includes only vague guidance on the geographic area to be reported on. Nevertheless, responses of residents within neighborhood clusters were aggregated to the boundaries of the cluster, introducing uncertainty as to the geographic validity of collective efficacy reports.^4^ Second, the aggregation procedure constrains collective efficacy to vary across neighborhood clusters (or tracts in some applications), precluding identification of within-cluster variation. Recent efforts have attempted to identify local variability in collective efficacy through dense sampling of small areas such as urban blocks (Weisburd et al. 2020, 2021) . Although this approach has yielded evidence of within-neighborhood variability in collective efficacy, its focus on residential areas and reporters highlights a third, critical set of concerns regarding extent approaches: (1) a lack of attention to mobility-induced nonresident presence in urban areas and the importance of “outsiders” in shaping the potential for collective control of crime, and (2) a neglect of collective efficacy measurement in commercial areas (which nonresidents routinely inhabit).

We draw on data from the Adolescent Health and Development in Context to describe and apply a measurement strategy that addresses these concerns while also offering a more cost-efficient approach than that introduced in the original PHDCN. Specifically, we collect multiple collective efficacy ratings of routine activity locations (including home) from a smaller sample of urban residents and capitalize on the location-level rating data to generate spatially continuous collective efficacy estimates varying at the point level. We employ local collective efficacy predictions derived from a Bayesian land use filtering approach that smooths collective efficacy reports, differentiating spatial dependency processes by land use type. This strategy acknowledges emerging evidence of potentially distinct informal social control processes by land-use type (Wickes et al. 2019; Corcoran et al. 2018) and the corresponding need for a more spatially targeted power-borrowing strategy for spatial processes that are heterogeneous in terms of the strength of spatial dependence and variability. In turn, continuous, fine scale predictions of collective efficacy allow us to address the question of within-neighborhood association between local collective efficacy and violence for a large urban area (as opposed to a selection of densely sampled micro-neighborhoods).

Point-level data on collective efficacy provide an opportunity to model effects on address-based estimates of violence (often available in administrative data on crime) using a point process framework. As noted, the now-established spatial concentration of crime within neighborhoods necessitates a better understanding of the social processes that may account for this within-neighborhood variability. Using an inhomogeneous Poisson point process model, we examine the association of local collective efficacy with violence independent of the effects of block group level collective efficacy. Our findings offer evidence of both negative associations of collective efficacy with violence at the block group level (consistent with decades of research) but at the point level (adjusting block group collective efficacy) as well. These results demonstrate the importance of ongoing research on the protective effects of collective efficacy and the determinants of location-level variation in this critical urban social process.

Finally, in exploratory models, we estimated coefficients for collective efficacy associations with violence by land-use type. These models should be considered highly exploratory but nevertheless highlight both the potential of our data collection and modeling approach to shed light on the within-neighborhood complexity of collective efficacy effects while also cautioning analysts to be sensitive to the impact of choices regarding covariates in the model for collective efficacy predictions.

In a research environment shifting toward the use of “Big Data” resources to proxy important social processes, we encourage researchers to consider original data collection using survey methods explicitly asking about these processes not just in home neighborhoods, but also in individuals’ routine mobility patterns. Such an approach – while by no means cost free – can be implemented in cost-effective ways to engender spatially fine-grained estimates of social processes (including, but beyond collective efficacy, such as physical and social disorder). Moreover, the emerging use of ecological momentary assessment (short surveys typically answered on a smartphone capturing aspects of the spatial environment in situ) to measure the dynamic nature of urban environments constitutes an important direction for research on social climates (Wickes et al. 2024; Cornwell and Goldman 2020). The Bayesian land use filtering approach applied here demonstrates the importance of capturing nuanced spatial dependence processes in generating estimates of these processes. These innovations hold the potential to substantially reinvigorate the measurement of urban social climate and its role in urban spatial inequality.

## Footnotes

1 As of 2018, Columbus is the 14th largest city in the US by population, with city and metro area populations of approximately 900K and 2.1M, respectively.

2 AHDC caregivers were asked to provide four street intersections or landmarks they “think of as the boundaries of [their] neighborhood” (Pinchak et al. 2021). To align with the routine activity location reports, we index these neighborhood reports in the land-use filtering model to respondents’ home addresses.

3 Comparisons are based on the Widely Applicable Information Criterion (WAIC), which is asymptotically equivalent to the cross validation loss (Watanabe 2010).

4 Note that the strategy employed here also incorporates a rating of “neighborhood” as a distinct location among the set of routine locations considered. Although the same concerns apply, the rating is treated as describing a point (location of residence) rather than an areal unit and offers significantly less influence on the overall predicted values of the collective efficacy dimensions.
